# BioPPSy: An Open-Source Platform for QSAR/QSPR Analysis

**DOI:** 10.1371/journal.pone.0166298

**Published:** 2016-11-10

**Authors:** Marta Enciso, Nastaran Meftahi, Michael L. Walker, Brian J. Smith

**Affiliations:** Department of Chemistry and Physics, La Trobe Institute for Molecular Science, La Trobe University, Victoria 3086, Australia; University of Minnesota Twin Cities, UNITED STATES

## Abstract

The reliability of quantitative structure-property relationship (QSPR) and quantitative structure-activity relationship (QSAR) models is often difficult to assess due to the problems of accessing the tools and data used to build the models. We present here BioPPSy, which aims to fill this gap by providing an easy-to-use open-source software platform. We demonstrate the program capabilities by calculating three key properties used in drug discovery, aqueous solubility, Caco-2 cell permeability and blood-brain barrier permeability. A comparison is made with a number of previously reported methods, taken from the literature, for each property. The software, including source code, current models and databases, is available from https://sourceforge.net/projects/bioppsy/.

## Introduction

The ability to identify *a priori* successful drug-like molecules from a plethora of possible candidates is a critical hurdle for the pharmaceutical industry in terms of time and resources [1]. To address this obstacle, *in silico* prediction of chemical properties has become an essential tool in the process of drug discovery and development [2]. Quantitative Structure-Property Relationship (QSPR) models are widely used to predict all relevant pharmacokinetics properties, particularly adsorption, distribution, metabolism, excretion and toxicity (commonly known as ADME/Tox properties).

QSPR methods are models that link a set of known variables (known as “descriptors”, which are related to the chemical structure of the molecules) to a certain property. A mathematical relationship between a set of descriptors and the property is established by fitting a *training set*, i.e. a group of molecules whose experimental property value is known. Thanks to their simplicity and good performance, many different QSPR models have emerged in recent years [3]. The utility of QSPR tools is largely affected by two factors: (a) their reliability, i.e. their ability to make predictions outside the training set, and (b) access to the tools, methods and experimental databases reported in the literature. These two factors are tightly connected, as limited access to raw data strongly hinders further improvement of the state of the art tools, particularly when it would be desirable to incorporate new experimental data into the process of model training.

**Fig 1:**
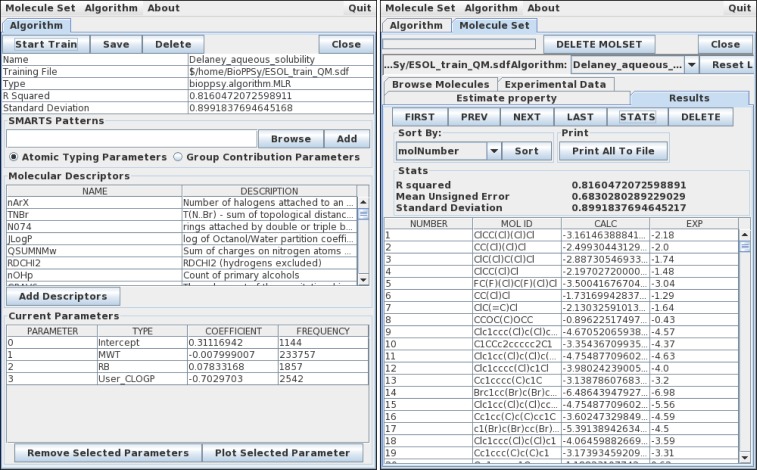
Snapshot of the BioPPSy software.

The BioPPSy software system addresses these problems by delivering an open-source tool set for performing QSPR analysis and providing access to the experimental data used to derive the models. BioPPSy presents an easy-to-use graphical interface (see Fig 1 for a snapshot). The software is programmed in Java and is freely available to use and modify. At the current stage of development several different analysis methods have been implemented, as well as a wide set of molecular descriptors.

The BioPPSy software is a **BIO**chemical **P**roperty **P**rediction **SY**stem. Here we show the capabilities of BioPPSy by predicting three critical ADME/Tox properties for drug discovery: aqueous solubility, Blood-Brain Barrier (BBB) permeability and Caco-2 cell permeability. The first property is arguably the most critical of any drug, as its solubility governs both the rate of dissolution of the compound and the maximum concentration reached in the gastrointestinal fluid[4]. As a result it determines whether the compound is orally available and can be ultimately delivered to its intended target [5].

Blood-brain barrier permeability (BBB) is a measurement of how easily a molecule can reach the brain from the general blood circulation; therefore BBB permeability is a central property not only for neurotherapeutics (where high BBB permeability is desirable) but also for other drugs that may be harmful for the brain [6]. Lastly, the Caco-2 cell line is one of the most widely utilised models of intestinal absorption, being key to estimate the bioavailability of a compound [7].

### BioPPSy workflow

**Fig 2:**
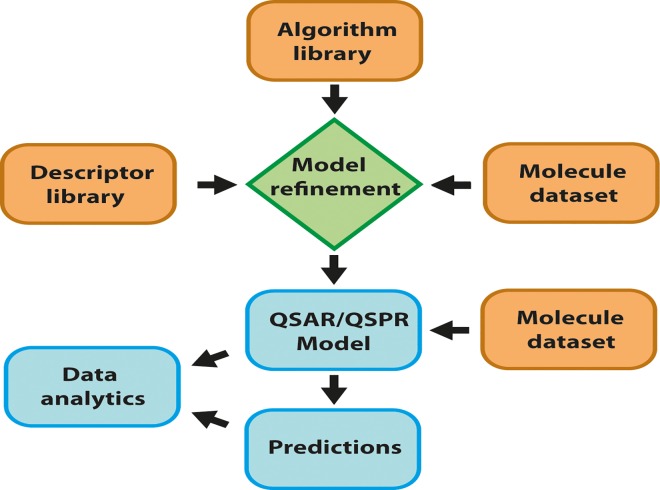
Workflow of the BioPPSy software.

The BioPPSy program has 2 main functionalities, creation of a QSPR/QSAR model and the prediction of properties using this model. The workflow involves the selection of a set of descriptors, an algorithm, and the input of a molecule data set. The model is created from this information, and stored for later use (Fig 2). The current version of the software includes ~100 descriptors–new descriptors can be included by placing Java scripts in the descriptor directory and including the name of the new descriptor in the descriptor list. Algorithms currently include MLR and other linear methods–non-linear methods will be included in the future. The molecule dataset must be provided in structure data format (sdf).

## Features

BioPPSy is designed to provide a simple and flexible tool for QSPR studies. It presents a user-friendly graphical interface which allows the user to build their own models. Thanks to its open-source Apache license, additional features can be included in the software using Java. The program uses Chimera software for molecule visualisation [8], which should be downloaded independently, and is partially based on JOELib, a freely available cheminformatics library [9].

The BioPPSy software completes the two main tasks of a QSPR software [10]: it builds QSPR models from a given training set, and uses previously derived models to make property predictions.

### Definition of a QSPR model

A QSPR model is a mathematical rule that calculates a property *P* (such as solubility, blood-brain barrier permeation or Caco-2 cell permeability) based on the values of a number of descriptors (*d*_*1*_, *d*_*2*_,…, *d*_*N*_) which can be computed from the molecular description, including 3-D structures, of the compounds. A new QSPR model can be set up in BioPPSy from the “Algorithm” menu.

The relationship between property and descriptors is established by using a set of compounds of known *P*, usually called the *training set*. The training sets used for the examples shown in this article can be found as part of the BioPPSy source code, although any other molecule set (in multi-SDF format) can be selected by the user. Given a training set, the data is fitted. There are many mathematical methods that can be used to perform this fitting [11]. BioPPSy has some of the most popular methods implemented, such as multivariate linear regression (MLR), mean centered algorithm and mean centered unit variance (MCUV). Some properties require more specific mathematical algorithms; as an example BioPPSy presents the Klopman algorithm for solubility calculations [12]. This method does not fit the property *P* (in this case, solubility) but its stereographic projection; this derived property is then fitted through a MCUV algorithm [12].

Regarding the choice of descriptors, BioPPSy can currently calculate more than 165 different descriptors. Some of them have been taken from the JOELib library of molecular descriptors [9] while others have been specifically implemented for BioPPSy, mostly following their description listed in the compendium by Todeschini & Consonni [13]. Other descriptors will be implemented in the future and can also be added to the source code by the user.

Once the method of fitting and descriptors have been chosen, the algorithm is trained. The quality of the obtained QSPR model can be automatically assessed through the software, as BioPPSy automatically displays the coefficient of determination (r^2^) and the standard deviation of the data (σ). In addition, a plot of *P* versus any of the used descriptors, *d*_*i*_, can be displayed. Following algorithm development, the algorithm can be saved for later use.

### Property prediction

The final aim of any QSPR software is property prediction. This can be done in BioPPSy through the “Molecule Set” menu. Any molecule data set in multi-SDF format can be uploaded and explored through BioPPSy, including structure visualization using the Chimera software [8] (see Fig 1). A property can be predicted provided there is a suitable algorithm, which can be selected from the BioPPSy options. If the data set already presents an experimental value for the property (for instance, in the case of a *test set*), the quality of the prediction is automatically evaluated for each compound in the data set.

## Results

We have used BioPPSy to predict three key properties for drug discovery, solubility, blood-brain barrier permeability and Caco-2 cell permeability. Many different QSPR methods have been proposed for each of these properties. It is often difficult to compare the predictive ability of the methods themselves, as they rely on different training and test sets, and have been generated using different software. In this Section we aim to carry out this comparison using the same training sets and the BioPPSy software. This guaranties that the only differences in performance are related to the models themselves, minimising additional and confounding variables.

### Aqueous solubility

Aqueous solubility has played a central role in *in silico* methods for drug discovery since the pioneering observation of Lipinski and co-workers regarding the importance of aqueous solubility in drug absorption [14]. They established the so-called “rule of five”, which relates the solubility of a given compound to several molecular properties. Since then, many different methods have been proposed [12,15–19]. We have selected several of these methods and re-derived them using BioPPSy. The main difference across the methods lies in the nature and number of descriptors used, and the origin and size of datasets used for training.

Klopman and Hou [12] used 118 group contribution descriptors; these descriptors refer to general atomic properties, functional groups and fragment-based characteristics. Their model was fitted using a training set of 1168 organic compounds. The model developed by McElroy and [15] uses a combination of 11 topological, geometric and electronic descriptors. They used a training set of 298 heteroatom-contaning organic compounds. Tetko *et al*. [16] proposed a linear QSPR model based on 33 descriptors linked to electrotopological characterstics that was fitted to a training set composed of 879 organic compounds. The Cheng and Merz [17] QSPR model is based on 8 descriptors that included the count of hydrogen bond donors and acceptors, the number of rotational bonds, and the water/octanol partition coefficient; these descriptors, which do not rely on the 3-D structure of the molecule, were used to fit a training set of 755 organic compounds. Delaney [18] proposed a method using just 4 descriptors, that also did not require a 3-D representation of the molecules, trained against several datasets, including one with 1144 small compounds and a larger one (contaning additional Syngenta propietary molecules); neither the coefficient of determination nor the standard deviation were reported for the small dataset, although, using the larger dataset a model with r^2^ = 0.69 and σ = 1.01 was produced. The last model considered here was the model proposed by Hou *et al*. [19], where 76 descriptors (based on the atom contribution approach) were used; the training set consisted of 878 organic compounds. In summary, we have explored 6 different QSPR models trained using between 4 and 118 descriptors on databases of different composition and sizes, ranging from 298 to 1168 compounds. A comparison of the methods, and the performance of BioPPSy to reproduce these models, is presented in Table 1. When creating these models, we have used MLR in all cases; this differs from the approach employed by Klopman and Hou who used MCUV with stereographic projection [12]. While this method is included in the BioPPSy package, it did not produce superior results than standard MLR.

**Table 1:** Comparison of performance of BioPPSy with literature methods for predicting the logarithm of aqueous solubility.

	*d*_*N*_	*N*	Literature^a^		BioPPSy^a^			BioPPSy^b^			
			r^2^	σ	r^2^	σ	Δ_max_	r^2^	σ	MUE	Δ_max_
Klopman and Hou. [12]	118	1168	0.95	0.50	-	-	-	0.73	1.05	0.80	-5.9
McElroy and Jurs [15]	11	298	0.79	na	0.74	0.94	3.4	0.70	1.11	0.85	3.9
Tetko *et al*. [16]	33	879	0.86	na	0.83	0.82	-3.0	0.83	0.84	0.66	-3.0
Cheng and Merz [17]	8	755	0.84	na	-	-	-	0.84	0.42	0.61	-4.4
Delaney [18]	4	1144	na	na	0.82	0.89	4.0	0.82	0.86	0.64	4.7
Hou *et al*. [19]	76	878	0.96	0.61	0.92	0.57	-2.2	0.90	0.64	0.50	-2.3

*d*_*N*_ is the number of descriptors used in each model (excluding intercept). *N* is the number of molecules in the datasets used in the original model. r^2^ is the coefficient of determination of the fitting and σ is the standard deviation. MUE is the mean unsigned error. Δ_max_ is the largest difference between experimental and predicted solubility. A dash (–) indicates the dataset was not available. ‘na’ indicates the coefficient of determination or standard deviation was not reported.

^a^Regression statistics obtained using *d*_*N*_ descriptors on datasets of size *N* reported in the literature.

^b^Regression statistics obtained using *d*_*N*_ descriptors on the dataset of 1297 organic compounds extracted from the AQUASOL and PHYSPROP datasets.

The regression analysis statistics from BioPPSy generally match closely the literature results, indicating that BioPPSy can reproduce QSPR calculations reported in the literature; differences in coefficient of determination are typically less than 0.05. Not unexpectedly, the coefficient of determination is better (closer to 1.0) the larger the number of descriptors. The largest difference between the results from BioPPSy and the literature data was for the Klopman and Hou model; notably, 7 of the 118 descriptors used by Klopman and Hou were not represented in the Huuskonen data set. Without access to the training set used by Klopman and Hou, we cannot identify the root cause of the difference in regression statistics.

To test the relative performance of these 6 different methods, we used a single data set complied by Huuskonen [22], formed by 1297 organic compounds extracted from the AQUASOL database of the University of Arizona [20] and the PHYSPROP database [21], to train models using the same set of descriptors used in each model. A comparison of the methods can be found in Table 1. The models created by Tetko *et al*. and Hou *et al*. used a subset of the Huuskonen set. The data sets used by McElroy and Jurs, and Delaney have 61% and 15% coverage, respectively, by the Huuskonen set. Discrepancies between the predictions from BioPPSy and the other methods could be attributed to the use of 3-D structures in BioPPSy to calculate shape-based properties, such as polar surface area; Delaney, for example, uses 1-D SMILES strings to predict the polar surface area.

Both the coefficient of determinations and standard deviations are remarkably similar to the results obtained using the smaller training datasets, indicating that the models are somewhat independent of the size and contents of the training dataset. Additionally, all models perform well, with r^2^ greater than 0.8 in most cases. Notably, the good performance of these models suggests that linear algorithms are reasonable methods for the prediction of aqueous solubility. Arguably, the better performaning methods are those from Hou *et al*. [19], which use group contribution descriptors, although this method also uses a very large number of descriptors.

The experimental (log) solubilites in the combined AQUASOL [20] and PHYSPROP [21] datasets cover a range of values from -11.6 to 1.6. The largest deviation between the experimental and predicted solubilities (Δ_max_) and the mean unsigned error (MUE) was considerably smaller using the Hou *et al*. model than the other models investigated.

### Brain blood barrier permeation

Blood brain barrier permeation measures the ability of a compound to reach the central nervous system, i.e. the brain uptake of the molecule. Computational QSPR models have been used to predict BBB transport since the mid-1990s [23]. QSPR methods for BBB prediction developed to date generally use descriptors that reflect two key aspects, molecular size and lipophilicity [24]. We have selected several models [23,25–27] and attempted to reproduce the results reported in the literature with BioPPSy. Additionally, we have trained these models using a multivariate linear regression algorithm and a significantly larger training set of 181 compounds compiled by Garg and Verma [28].

The selected models use linear algorithms and differ in the number and characteristics of the descriptors used, as well as the size and nature of their training sets. Kansy and van der Waterbeemd [23] proposed a model based on only two descriptors (polar surface area and volume) and trained the model with a data set of just 20 compounds. Hou and Xu [25] related the blood-brain barrier permeation with four descriptors, octanol/water partition coefficient, PNSA2, number of rotatable bonds and radius of gyration, fitted to a training set of 59 compounds. The model proposed by Clark [26] used the polar surface area and octanol/water partition coefficient as descriptors and a training set of 55 compounds. Feher [27] developed a model using 3 descriptors, polar surface area, octanol/water partition coefficient and number of hydrogen bond acceptors, and a training set of 61 compounds. In summary, these models for log BBB prediction used very few descriptors, but were applied to rather small training sets. A comparison of the results reported in the literature and those obtained using BioPPSy is presented in Table 2, along with the results for each method trained against the larger dataset of Garg and Verma [28].

**Table 2:** Comparison of performance of BioPPSy with literature methods for predicting the logarithm of blood-brain barrier permeability.

	*d*_*N*_	*N*	Literature^a^		BioPPSy^a^			BioPPSy^b^			
			r^2^	σ	r^2^	σ	Δ_max_	r^2^	σ	MUE	Δ_max_
Kansy and van de Waterbeemd [23]	2	20	0.70	0.45	-	-	-	0.45	0.54	0.42	-1.68
Hou and Xu [25]	4	59	0.76	0.41	-	-	-	0.49	0.53	0.25	-1.52
Clark [26]	2	55	0.77	0.46	0.75	0.82	-1.42	0.52	0.51	0.38	-1.71
Feher *et al*. [27]	3	61	0.73	na	0.63	0.39	-1.17	0.54	0.50	0.38	1.58

^a^Regression statistics obtained using *d*_*N*_ descriptors on datasets of size *N* reported in the literature.

^b^Regression statistics obtained using *d*_*N*_ descriptors on a dataset containing 181 compounds [28].

We were able to reproduce the results reported by Clark *et al*. [26] and Feher *et al*. [27] with the original datasets used in these studies; this was not possible for the remaining methods because the original datasets used in these studies were not available. BioPPSy produces equivalent predictions in conditions similar to those reported previously. In stark contrast, when applying these algorithms to a much larger dataset (roughly 3–times the size of the datasets used previously) the performance is significantly poorer; in these cases the correlation between descriptors and the experimental property reduces from r^2^~0.7, reported using the smaller dataset, to r^2^~0.5 with the larger dataset. The Garg and Verma [28] data set covers 36% and 12%, respectively, of the Clark and Feher data sets, and thus represents a significant variation on the training set over the original models. The compilation of log BBB by Garg and Verma covered the range -2.2 to 1.5; the calculated mean unsigned errors from all models is significant. This dramatic reduction in performance of the model highlights the necessity of using a broad training set in developing a QSPR model. The origins of the large discrepancies between reported and calculated data can be attributed to the small size of the training set [29], stressing the need of additional descriptors and more complex models.

### Caco-2 cell permeability

Caco-2 cell permeability is routinely computed in drug development studies as a surrogate for intestinal absorption [7]. We have explored the performance of several methods from the literature [30–34]; the results are presented in Table 3. Each of the methods employ a small number of molecular and topological descriptors; in some cases only a single descriptor has been used (for example, the topological surface area [32] or polar surface area [33]) while other methods use a combination of them (polar surface area and molecular weight [30] or hydrogen bond-related properties and the octanol-water partition coefficient [31]). A slightly different approach is used by Gozalbes *et al*. [34], where a combination of thirteen different descriptors (mostly based on atomic group types and charge-related group types) is used. We have implemented these descriptors in BioPPSy to further analyse the software performance. A training set of 159 compounds has been used, taken from the training, validation and external datasets of Gozalbes *et al*. [34]. In all cases we have used multivariate linear regression algorithms to obtain the results presented in Table 3.

**Table 3:** Comparison of performance of BioPPSy with literature methods for predicting the logarithm of Caco-2 cell permeability.

	*d*_*N*_	*N*	Literature^a^		BioPPSy^a^			BioPPSy^b^			
			r^2^	σ	r^2^	σ	Δ_max_	r^2^	σ	MUE	Δ_max_
Ertl *et al*. [32]	1	9	0.98	na	0.96	0.22	-0.32	0.31	0.71	0.61	-2.67
Palm *et al*. [33]	1	6	0.99	na	0.96	0.15	0.15	0.23	0.78	0.65	-2.89
Osterberg & Norinder [31]	4	11	0.92	0.21	0.99	0.04	0.07	0.39	0.70	0.56	-2.39
van de Waterbeemd *et al*. [30]	2	17	0.69	na	0.65	0.61	-1.12	0.24	0.78	0.65	-2.88
Gozalbes *et al*. [34]	13	97	0.77	0.49	0.70	0.50	-1.31	0.58	0.58	0.45	-1.68

^a^Regression statistics obtained using *d*_*N*_ descriptors on datasets of size *N* reported in the literature.

^b^Regression statistics obtained using *d*_*N*_ descriptors on the 159 compound dataset of Gozalbes *et al*. [34].

The results using the Gozalbes *et al*. data set showed large discrepancies with the originally reported data, as had been observed for the prediction of blood brain barrier permeation. The main source of this variance comes again from the very different sizes of the data sets, with the data set compiled by Gozalbes *et al*. being up to 25-times larger than some of those used in the earlier work. The predictions using BioPPSy were comparable to those reported by Gozalbes *et al*. [34]. Critically, using the data sets used in the original work of the other methods listed in Table 3 we were able to obtain very similar coefficients of determination (r^2^) as those reported. The impact of the database size on the accuracy and applicability of QSPR models is widely appreciated, and had been discussed in relation to Caco-2 cell permeability in particular in the original work of Gozalbes *et al*. [34]. The Gozalbes data set of the logarithm of the Caco-2 cell permeability covers the range -7.6 to -3.7; the average mean unsigned error from all 5 models of 0.58 represents 15% of the experimental range, and reflects the most optimistic accuracy that can be obtained from these models. In all models, the largest error was associated with the same molecule, 2-(1-(aminomethyl)cyclohexyl)acetic acid, normally zwitterionic at neutral aqueous pH. None of the models examined here include specific descriptors that define a zwitterion.

### Summary and Conclusions

Two of the main challenges of QSPR modelling are the access to raw data and the reliability of the models reported in the literature. These two issues can be partly solved by the use of open-source software and datasets. BioPPSy is an open-source software that provides an easy-to-use interface for QSPR modelling. At the current stage it presents a reasonably wide selection of descriptors as well as several linear algorithms, which will be extended in the future to other linear and non-linear algorithms.

We have studied several QSPR models for three key properties in drug development, aqueous solubility, Caco-2 cell permeability and brain-blood barrier permeation. In all cases we have used resonably large datasets already reported in the literature (also available through BioPPSy source code) and the functionalities currently implemented in the software. In the first case, aqueous solubulity, we have obtained a very good agreement between the models produced using BioPPSy and the literature models.

In the latter two cases, Caco-2 cell permeability and brain-blood barrier permeation, however, larger differences were observed. The source of these differences could be linked to the sizes of the training sets used; the larger and more diverse data sets could not be modelled accurately with a limited number of descriptors. Caco-2 cell permeability results still correlated well using the descriptors of the earlier methods but the BBB data presented only low-to-moderate correlations, indicating that further model development is needed.

We have limited our review of earlier models to those derived using MLR. These models are plagued by issues of overfitting, yet MLR remains ubiquitous in the field, and is the principle reason why we focused on this particular approach of QSPR. We are currently expanding the capabilities of the program to include more contemporary methods (in particular non-linear methods) that will permit a comparison across a variety of different approaches.

A number of on-line web applications (eg. ochem [35], Chembench [36]) and open-source programs (eg. ChemmineR [37] and CDK [38]) with functionalities similar to BioPPSy have been developed. The BioPPSy platform offers several advantages: (1) both the datasets employed in model generation and source-code are available, (2) BioPPSy is not only a library, but a full working program, and (3) Java is a user-friendly language that makes further development by any user simple and straightforward.
